# Metabolic Disorders and Diabetic Complications in Spontaneously Diabetic Torii *Lepr* ^*fa*^ Rat: A New Obese Type 2 Diabetic Model

**DOI:** 10.1155/2013/948257

**Published:** 2013-02-25

**Authors:** Yusuke Kemmochi, Kenji Fukui, Mimi Maki, Shuichi Kimura, Yukihito Ishii, Tomohiko Sasase, Katsuhiro Miyajima, Takeshi Ohta

**Affiliations:** Japan Tobacco Inc., Central Pharmaceutical Research Institute, 1-1, Murasaki-cho, Takatsuki, Osaka 569-1125, Japan

## Abstract

Spontaneously Diabetic Torii *Lepr* ^*fa*^ (SDT fatty) rat, established by introducing the *fa* allele of the Zucker fatty rat into SDT rat genome, is a new model of obese type 2 diabetes. Both male and female SDT fatty rats show overt obesity, and hyperglycemia and hyperlipidemia are observed at a young age as compared with SDT rats. With early incidence of diabetes mellitus, diabetic complications, such as nephropathy, retinopathy, and neuropathy, in SDT fatty rats were seen at younger ages compared to those in the SDT rats. In this paper, we overview pathophysiological features in SDT fatty rats and also describe new insights regarding the hematology, blood pressure, renal complications, and sexual dysfunction. The SDT fatty rats showed an increase of leukocytes, especially the monocyte count, prominent hypertension associated with salt drinking, end-stage renal disease with aging, and hypogonadism. Unlike other diabetic models, the characteristic of SDT fatty rat is to present an incidence of diabetes in females, hypertension, and retinopathy. SDT fatty rat is a useful model for analysis of various metabolic disorders and the evaluation of drugs related to metabolic disease.

## 1. Introduction

Type 2 diabetes mellitus is a polygenic disorder that is caused by a metabolic and/or hormonal imbalance between insulin secretion from *β* cells and insulin sensitivity in peripheral tissues, both of which might be modified by genetic and environmental factors [1]. The decreased sensitivity to insulin leads to an increased requirement for insulin and is often associated with obesity in which metabolic disturbances are marked in insulin-target organs, such as the liver, muscle, and adipose tissues [2]. Obesity plays key roles in the pathophysiology of several metabolic diseases and is a risk factor for diabetes mellitus and for dyslipidemia [3]. Diabetic animal models play critical roles in the elucidation of the mechanisms of diabetes mellitus and the complications [4] and in the development of novel drugs as treatments [5, 6]. Based on the previously mentioned concept, a novel model of obesity-related diabetes was established by Masuyama et al. [7]. They established a congenic line of the Spontaneously Diabetic Torii (SDT) rat by introducing the *fa* allele of the Zucker fatty rat into the SDT rat genome via the Speed Congenic Method using a PCR technique with DNA markers. This congenic strain has been maintained by intercrossing between *fa*-heterozygous littermates. SDT rats develop diabetes independent of obesity [8–10]. They have normal body weights, blood glucose levels, insulin levels, and lipid levels until about 16 weeks of age and, thereafter, develop hyperglycemia associated with hypoinsulinemia, which results from the degeneration of pancreatic beta cells [11–13]. As a result of chronic hyperglycemia, the SDT rats develop profound complications in eyes, peripheral nerves, kidneys, and bones [8, 9].

## 2. Physiological Features

**Figure 1:**
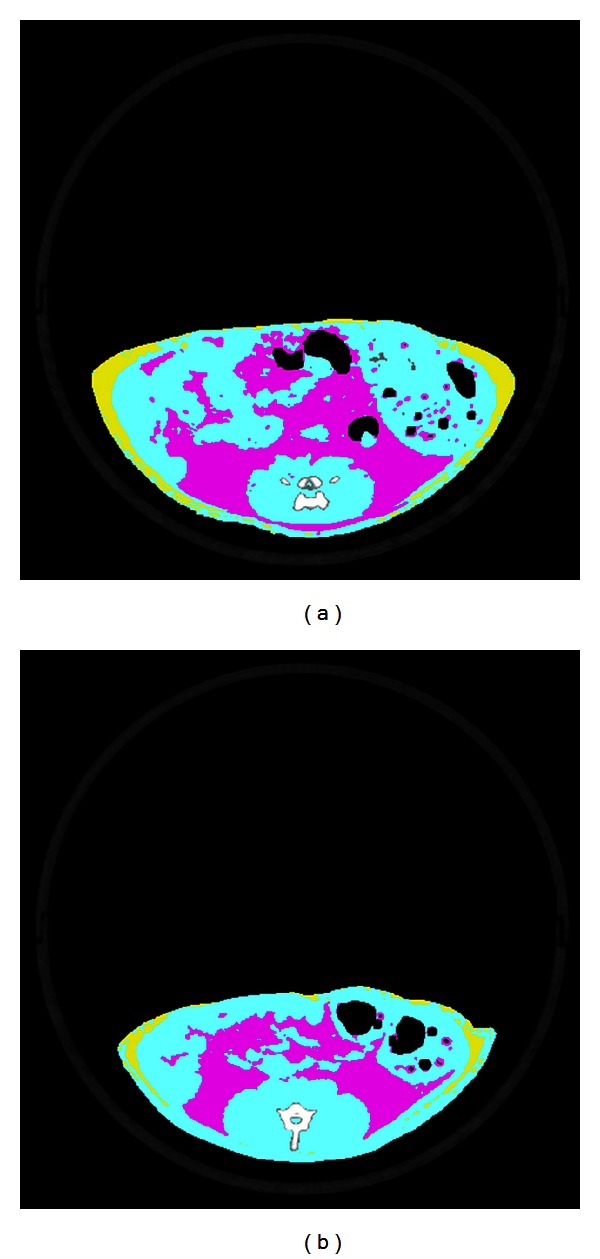
Computed tomography analysis at 12 weeks of age (representative tomogram). (a) SDT fatty rat; (b) SDT rat. Yellow: subcutaneous fat; red: visceral fat; blue: lean [14].

The *fa/fa* (SDT fatty) rats of both sexes became overtly obese and showed a significant hyperphagia. Also, the BMI (body mass index) was greater in SDT fatty rats (mean value, 0.91 and 0.87 g/cm^2^ in males and females, resp.) than that in lean rats (0.75 and 0.58 g/cm^2^ in males and females, resp.) at 14 weeks of age [7]. Fat weights in SDT fatty rats were higher than those in SDT rats. Both visceral and subcutaneous fat weights were significantly higher in SDT fatty rats, but, especially, the visceral fat weight was markedly elevated with aging (Table 1 and Figure 1) [14].

Serum glucose levels in SDT fatty rats of both sexes were elevated from 6 weeks, and lipid parameters such as serum triglyceride and total cholesterol levels in the rats were elevated from 4 weeks of age. The hyperglycemia and hyperlipidemia were sustained for a long time afterwards [15, 16]. The male SDT fatty rats showed hyperinsulinemia from 4 to 8 weeks of age, but after 16 weeks their insulin levels decreased to levels similar to those in SDT rats. In the female rats, hyperinsulinemia was shown from 4 to 12 weeks of age, and the insulin levels decreased gradually. Also, a remarkable rise in renal parameters such as urine volume and urine protein was shown in SDT fatty rats of both sexes.

**Figure 2:**
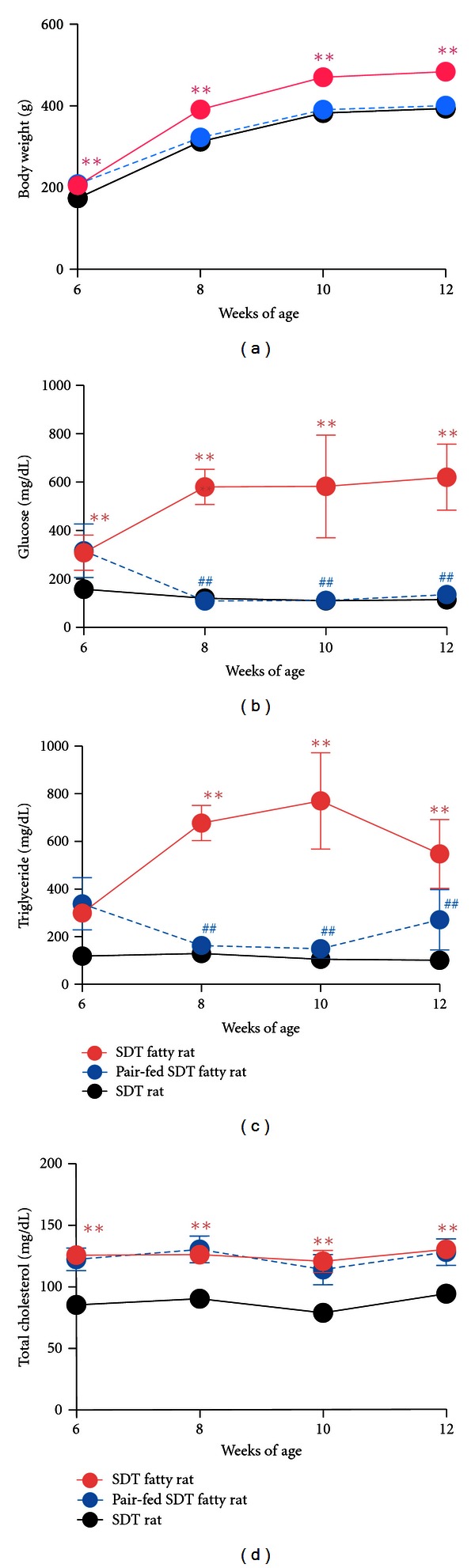
Changes in body weights (a) and serum parameters (b) glucose; (c) triglyceride; (d) total cholesterol) in SDT fatty rats, pair-fed SDT fatty rats, and SDT rats. Data represent mean ± standard deviation (*n* = 6). ^##^ *P* < 0.01: significantly different from SDT fatty rat. **P < 0.01: significantly different from SDT rat.

Effect of food restriction in SDT fatty rats was investigated [14, 17]. SDT fatty rats were subjected to pair feeding with SDT rats. Body weights of the pair-fed rats were similar with those of SDT rats. Improvement of hyperglycemia or hypertriglyceridemia was observed, but hypercholesterolemia was not entirely improved (Figure 2). The changes in adipose tissue were interesting. The visceral/subcutaneous (V/S) fat ratio decreased in the pair-fed rats (mean ± standard deviation: control rats, 2.04 ± 0.66; Pair-fed rats, 1.28 ± 0.19), although the total fat (visceral fat and subcutaneous fat) weight did not change (mean ± standard deviation: control rats, 137.5 ± 52.5 g; Pair-fed rats, 135.2 ± 10.4 g). Cell size of the epididymal fat in the pair-fed rats tended to decrease, and glucose oxidation level in epididymal fat in the pair-fed rats was recovered to a similar level with that in SDT rats [14].

**Figure 3:**
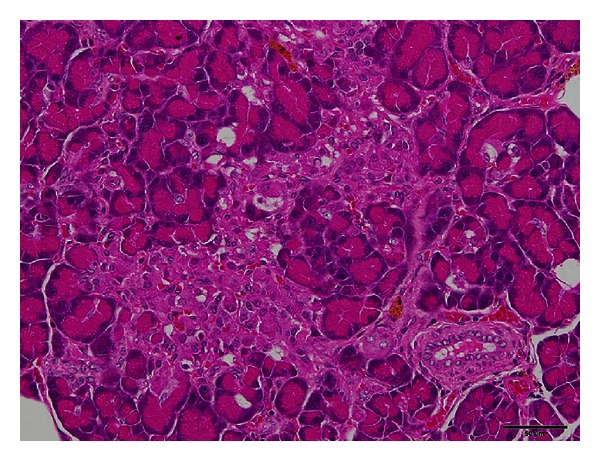
Histological analysis, HE stain. Pathological findings such as atrophy and fibrosis in pancreatic islets of female SDT fatty rat at 24 weeks of age. Bar = 50 *μ*m.

In the glucose tolerance test conducted at 9 weeks of age, SDT fatty rats showed higher serum glucose levels after glucose loading without any response of plasma insulin [18]. Those impaired glucose tolerance and insulin secretion were deteriorated with aging. In pancreatic islets of female SDT fatty rats, pathological findings such as vacuolation, hypertrophy, and hemorrhage were observed from 8 weeks of age, and findings such as atrophy and fibrosis in the islets were observed from 24 weeks of age (Figure 3) [16]. In SDT rats, glucose intolerance was observed in prediabetic stage [12, 13]. The histological features in the pancreas of SDT rats were as follows: in 10–20 weeks of age, slight changes such as hemorrhage, hemosiderin deposition, inflammatory cell infiltration, and fibrosis in and around the islets; in 25 weeks of age, hemosiderin deposition, cellular infiltration with lymphocytes and macrophages, and fibrous tissue proliferation in and around the islets [8].

New results in hematologic analysis are shown in Table 2. Nonfasted serum parameters, such as leukocyte count (WBC), erythrocyte count (RBC), hemoglobin (Hb), and hematocrit (Ht) level, were examined at 6 and 12 weeks of age. Also, differential counts of leukocytes, such as neutrophils, eosinophils, basophils, monocytes, and lymphocytes, were determined, respectively. Blood samples were collected from the tail vein of rats. The levels were measured using an automatic analyzer (ADVIA 120 Hematology System (Siemens AG), Erlangen, Germany). WBC and Ht levels in SDT fatty rats were significantly higher as compared with those in SD rats at both 6 and 12 weeks of age. In differential counts of leukocytes, the monocyte count was significantly higher at 12 weeks of age in SDT fatty rats. It is reported that type 2 diabetes mellitus or obesity is a chronic inflammatory state aggravated by factors that promote inflammation at the level of vasculature and adipose tissue [19–21]. Since the monocyte count in WBC is elevated, in the future, it is necessary to investigate the inflammatory state in SDT fatty rats. RBC and Hb levels in SDT fatty rats were comparable to those in SD rats at 6 and 12 weeks of age.

We examined blood pressure, known to be a risk factor for metabolic syndrome, in SDT fatty rats. In male SDT fatty rats, blood pressure was significantly higher from 8 to 24 weeks of age, as compared with age-matched SD rats [22]. Furthermore, we confirmed a new insight regarding blood pressure in SDT fatty rats: a high sensitivity to sodium. Blood pressure in SDT fatty rats is markedly elevated with sodium drinking. A 1% NaCl solution was given to male SDT fatty rats for 8 weeks, from 4 to 12 weeks of age. The systolic blood pressure in SDT fatty rats was significantly higher as compared with that in control-SDT fatty rats (mean ± standard deviation: NaCl-SDT fatty rats, 200.2 ± 22.0 mmHg; control-SDT fatty rats, 137.2 ± 13.0 mmHg). SDT fatty rats showed a high sensitivity for sodium. In other words, an elevation of blood pressure in the rats was more prominent after sodium loading.

## 3. Diabetic Complications

### 3.1. Microvascular Disease

**Figure 4:**
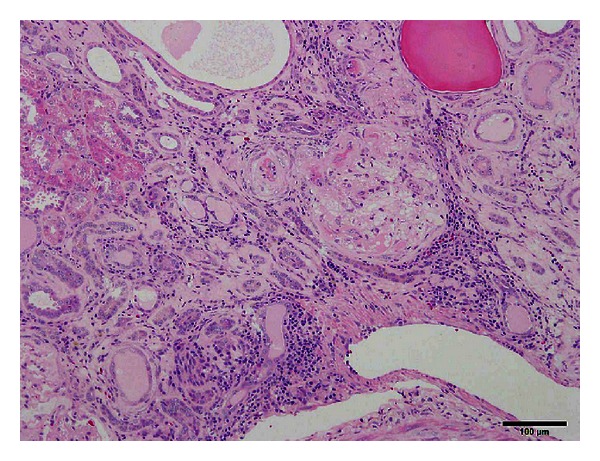
Histological analysis. HE stain. Severely progressed glomerular and tubulointerstitial lesions in male SDT fatty rat at 60 weeks of age There are completely sclerotic glomeruli and atrophic tubules with many inflammatory cells in the interstitum. Bar = 100 *μ*m.

With early incidence of diabetes mellitus, diabetes-associated complications in SDT fatty rats were seen at younger ages compared to those in the SDT rats. In male SDT fatty rats, histopathological examination of the kidneys revealed changes in the glomeruli from 16 weeks and in the renal tubules from 8 weeks of age [15]. In the glomeruli, glomerulosclerosis was observed from 16 weeks of age, and the sclerosis progressed with aging. Nodular lesions were observed at 40 weeks of age. In the renal tubules, glycogen deposition in the tubular epithelium (Armanni-Ebstein lesions) and tubular dilation were noted from 8 weeks of age, and the change progressed from 8 to 16 weeks of age. In female SDT fatty rats, a qualitatively equal change was observed in histopathological findings of kidneys [16]. The female rats revealed changes in the glomeruli from 32 weeks of age, and in the renal tubules from 16 weeks, and the changes progressed with aging. Furthermore, we investigated histopathological characteristics of the kidneys in male and female SDT fatty rats at 60 weeks of age. Diffuse glomerulosclerosis, including increased mesangial matrix and glomerular hypertrophy, was severely progressed in the SDT fatty rats. Moreover, tubular and interstitial lesions, including fibrosis and inflammatory cell filtration, were progressed in the SDT fatty rats (Figure 4). There were no clear sex differences in the morphological characteristics of the renal lesions.

Histopathological findings in lens, including hyperplasia of epithelium, vacuolation of fiber, and occurrence of Morgagnian globules, were observed from 8 weeks of age in male SDT fatty rats, and these changes progressed with aging. The female rats showed similar changes from 16 weeks of age. Also, retinal lesions, such as folding and thickening, were observed with aging in male and female SDT fatty rats.

**Figure 5:**
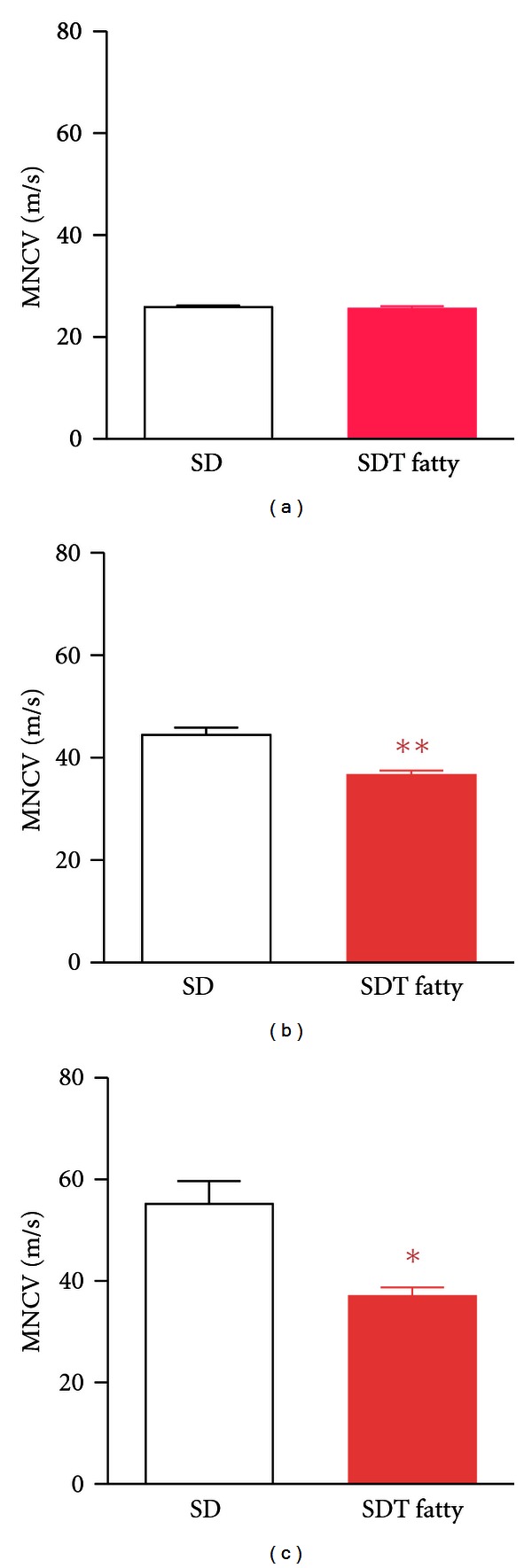
Changes in MNCV at 8 (a), 24 (b), and 40 (c) weeks of age in SD rats and SDT fatty rats. Data represent mean ± standard deviation (*n* = 5). *P < 0.05, **P < 0.01: significantly different from SD rat.

Diabetic peripheral neuropathy was evaluated at 8, 24, and 40 weeks of age in male SDT fatty rats. Tail motor nerve conduction velocity (MNCV) in the SDT fatty rat was delayed at 24 weeks of age and was further decreased at 40 weeks of age (Figure 5) [23]. Histopathologically, at 40 weeks of age, the fiber number was significantly decreased, and SDT fatty rats revealed significant atrophy in myelinated nerve. Six-week treatment of pioglitazone, a peroxisome proliferator-activated receptor (PPAR)-*γ* agonist, lowered blood glucose level and prevented delay of sciatic MNCV in SDT fatty rats [23].

### 3.2. Osteoporosis

**Figure 6:**
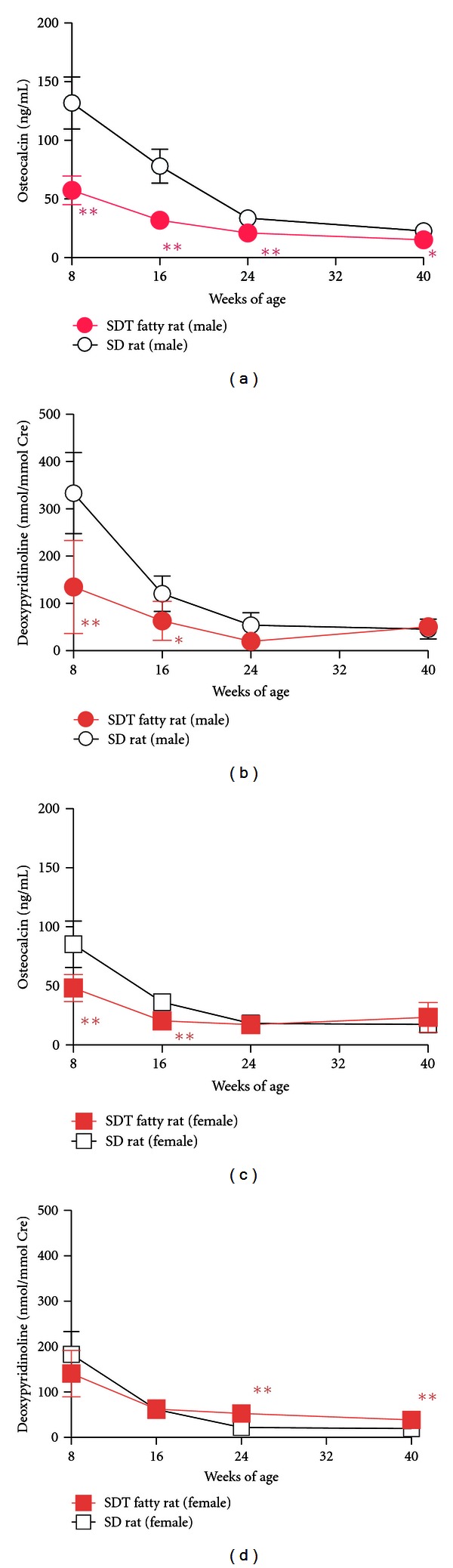
Changes in serum osteocalcin (a), (c) and urine deoxypyridinoline (b), (d) in SD rats and SDT fatty rats. Data represent mean ± standard deviation (Male: *n* = 5, Female: *n* = 10). *P < 0.05, **P < 0.01: significantly different from SD rat.

**Figure 7:**
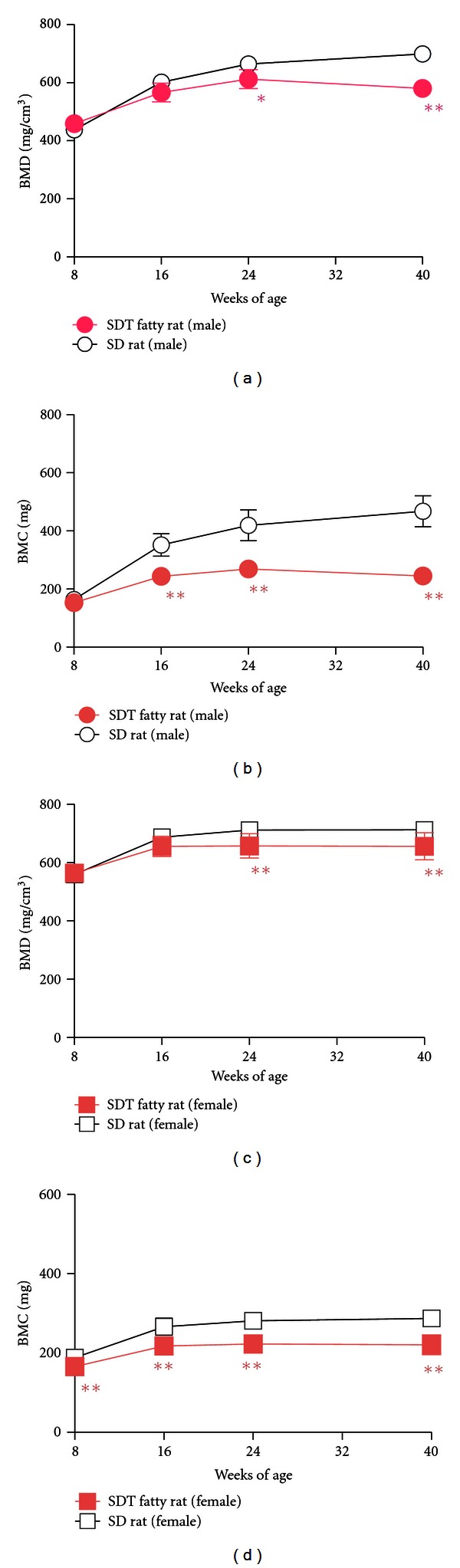
Changes in BMD (a), (c) and BMC (b), (d) in SD rats and SDT fatty rats. Data represent mean ± standard deviation (Male: *n* = 5, Female: *n* = 10). *P < 0.05, **P < 0.01: significantly different from SD rat.

We investigated the effects of obese type 2 diabetes on bone turnover, bone mass, and bone strength in SDT fatty rats [24, 25]. Both serum osteocalcin, a bone formation marker, and urine deoxypyridinoline, a bone resorption marker, levels were lower in male SDT fatty rats compared to age-matched SD rats from 8 to 40 weeks of age (Figures 6(a) and 6(b)). The early onset of diabetes and obesity in male SDT fatty rats induced decreases in both serum osteocalcin and urine deoxypyridinoline and resulted in low bone turnover at a young age, 8 weeks of age. Male SDT fatty rats showed lower bone mineral density (BMD) and bone mineral content (BMC) of the whole tibia (Figures 7(a) and 7(b)) and shortening of the tibia and femur compared to age-matched SD rats [25]. Deterioration in bone geometrical properties of the femur midshaft, such as cortical thickness and minimum moment of inertia, was observed in male SDT fatty rats. Furthermore, trabecular bone volume of the distal femur was lower in the SDT fatty rats. These negative effects on bone in the SDT fatty rats caused severe decreases in maximum load, stiffness, and energy absorption of the femur. In addition, serum levels of homocysteine, a candidate bone fragility marker, were elevated in male SDT fatty rats compared to age-matched SD rats (mean ± standard deviation: SDT fatty rats, 14.4 ± 3.4 nmol/mL; SD rats, 6.6 ± 1.7 nmol/mL). We also investigated changes in bone metabolism and bone quantity at 10, 15, 25, and 40 weeks of age in female SDT fatty rats [24]. Serum osteocalcin and urine deoxypyridinoline levels were lower at 10 weeks of age in the SDT fatty rats compared to those in SD rats, but only the urine deoxypyridinoline levels were elevated in the SDT fatty rats from 25 weeks of age (Figures 6(c) and 6(d)). Female SDT fatty rats showed lower BMC and BMD of the whole tibia from 8 to 25 weeks of age (Figures 7(c) and 7(d)). SDT fatty rat is a useful model to investigate bone abnormalities in obese type 2 diabetes.

### 3.3. Sexual Dysfunction

**Figure 8:**
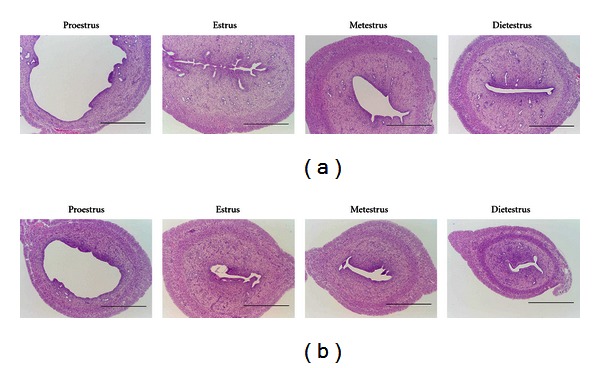
Histological analysis of uterus, HE stain. (a) SD rats and (b) SDT fatty rats at 12 weeks of age. Bar = 1 mm [26].

**Figure 9:**
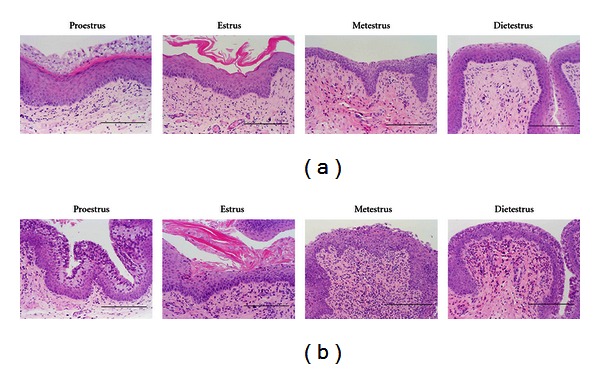
Histological analysis of vagina, HE stain. (a) SD rats and (b) SDT fatty rats at 12 weeks of age. Bar = 200 *μ*m [26].

Since SDT fatty rats (*fa*-homozygous) are infertile in both males and females, we used the SDT-*fa*/+ rats (*fa*-heterozygous) for reproduction. The reason the rats show infertility is unknown, but some functional disorders related to reproduction are observed [26]. We monitored the estrus cycle by vaginal smear cytology between 12 and 17 weeks of age and compared them with normal SD rats used as control. SD rats showed a regular 4-day estrus cycle, whereas SDT fatty rats showed an irregular 4- to 5-day estrus cycle with persistent estrus stage and metestrus extension. A large number of leukocytes appeared in smear of metestrus stage. Weight of reproductive organs (ovary, uterus, and vagina) in each estrus cycle stage was measured at 12 weeks of age, and the organs were examined by histopathological analysis. Relative weights of ovary, uterus, and vagina of SDT fatty rats were significantly lower than those of SD rats. In SDT fatty rats, histopathological changes, such as atrophy in uterus and inflammation in vagina, were observed (Figures 8 and 9). Irregular estrus cycle, increased leukocytes in vagina, or dysgenesis of reproductive organs might cause the infertility in female SDT fatty rats. Moreover, testosterone levels in male SDT fatty rats tended to be lower as compared with those in SD rats at 8 and 24 weeks of age (mean ± standard deviation: SDT fatty rats, 212.2 ± 84.4 pg/mL; SD rats, 960.4 ± 802.7 pg/mL; at 8 weeks of age, resp.). Since the testosterone levels in male SDT fatty rats are low, the reproductive function is considered to be decreased. In histological analysis of testis in the SDT fatty rats, we observed no change until 24 weeks of age. In further study, it is necessary to elucidate the mechanism of hypogonadism in SDT fatty rats.

## 4. Supply System of SDT Fatty Rat

In 2004, Dr. Masuyama and Dr. Shinohara (Research Laboratories of Torii Pharmaceutical Co., Ltd., Japan) established the congenic type 2 diabetes model Spontaneously Diabetic Torii fatty (SDT fatty) rat by introducing the *fa* allele of the Zucker fatty rat into the genome of the original SDT rat. SDT fatty rats were introduced to Central Pharmaceutical Research Institute, Japan Tobacco Inc. (Japan) and were characterized in detail by Dr. Ohta and Dr. Sasase. CLEA Japan has received right of production and sales from Japan Tobacco Inc. and has distributed the animals as SDT fatty rats since 2012.

## 5. Conclusion

Diabetes mellitus and diabetic complications in SDT fatty rats were found at a younger age than those in SDT rats. The early onset of diabetes or diabetic complication has advantages for the use of SDT fatty rats in diabetes research. Furthermore, not only the male rats but also the female rats developed diabetes mellitus at a young age. Female SDT fatty rat has the potential to become an important animal model of type 2 diabetes mellitus with obesity, especially in women, where few models currently exist. Since SDT fatty rats showed a hypertension with obesity, hyperglycemia, and hyperlipidemia, the rat might have potency to be established as a metabolic syndrome model. Also, it is interesting that SDT fatty rats showed various diabetic complications, such as osteoporosis and hypogonadism, with microangiopathy. Use of SDT fatty rats will assist in the further elucidation of the pathogenesis of human diseases related to metabolic disorder and in discovery of new drugs.

## Figures and Tables

**Table 1:** Changes of fat tissue weights in SDT fatty rats and SDT rats. Visceral and subcutaneous fat tissue weights were determined by computed tomography analysis. Data represent mean ± standard deviation (*n* = 6).

	4 weeks of age	12 weeks of age	20 weeks of age
SDT fatty rat			
Visceral fat (g)	6.3 ± 1.2**	54.4 ± 4.0**	74.1 ± 3.9**
Subcutaneous fat (g)	10.2 ± 1.5**	37.8 ± 6.2**	31.8 ± 4.4**

Total fat (g)	16.5 ± 2.7**	92.2 ± 9.9**	105.9 ± 7.8**

SDT rat			
Visceral fat (g)	2.1 ± 0.8	21.7 ± 3.4	25.1 ± 8.5
Subcutaneous fat (g)	0.9 ± 0.6	8.6 ± 1.3	10.2 ± 5.9

Total fat (g)	3.0 ± 0.5	30.3 ± 4.6	35.3 ± 14.4

**P < 0.01: significantly different from age matched SDT rats.

**Table 2:** Hematological examination of SD rats and SDT fatty rats. Data represent mean ± standard deviation (*n* = 5).

	WBC (×10^3^/*μ*L)	RBC (×10^6^/*μ*L)	HGB (g/dL)	HCT (%)	Neutrophil (%)	Lymphocyte (%)	Monocyte (%)	Eosinophil (%)	Basophil (%)
6 weeks of age									

SD	13.72 ± 2.21	6.44 ± 0.51	12.4 ± 2.3	42.5 ± 1.1	21.7 ± 5.4	73.5 ± 5.5	2.7 ± 0.5	1.5 ± 0.4	0.1 ± 0.0
SDT fatty	18.82 ± 2.43**	6.94 ± 0.20	13.5 ± 0.5	48.2 ± 1.7**	16.7 ± 3.0	78.6 ± 3.6	3.0 ± 0.7	0.8 ± 0.2**	0.2 ± 0.0

12 weeks of age									

SD	14.77 ± 2.15	8.30 ± 0.47	14.4 ± 1.3	47.6 ± 1.3	26.1 ± 6.3	69.4 ± 6.5	1.9 ± 0.6	2.2 ± 0.8	0.1 ± 0.1
SDT fatty	24.56 ± 1.77**	8.67 ± 0.58	15.8 ± 1.1	55.0 ± 4.3*	25.4 ± 3.4	68.9 ± 4.3	3.4 ± 1.0*	1.5 ± 0.1	0.2 ± 0.1

*P < 0.05, **P < 0.01: significantly different from age-matched SD rats.
